# Efficacy and safety of immunotherapy combined with chemoradiotherapy in limited-stage small cell lung cancer: a systematic evaluation and meta-analysis of randomized controlled trials

**DOI:** 10.3389/fimmu.2026.1731036

**Published:** 2026-07-22

**Authors:** Yixuan Wang, Yiyan Liu, Ning Zhan, Ke Jin, Lishuang Qi, Shilong Liu

**Affiliations:** 1 Department of Thoracic Radiation Oncology, Harbin Medical University Cancer Hospital, Harbin, China; 2 College of Bioinformatics Science and Technology, Harbin Medical University, Harbin, China; 3 Department of Radiation Oncology, Xiang’an Hospital of Xiamen University, Xiamen, China

**Keywords:** small cell lung cancer (SCLC), chemoradiotherapy (CRT), immunotherapy, programmed death 1 (PD-1), programmed death-ligand 1 (PD-L1)

## Abstract

**Background:**

Concurrent chemoradiotherapy (cCRT) remains the standard treatment for patients diagnosed with limited-stage small-cell lung cancer (LS-SCLC); however, recurrence rates remain high and prognosis remains poor. This study collected data regarding patient-reported outcomes and adverse events (AEs) to evaluate the effects of chemoradiotherapy combined with immunotherapy in patients with LS-SCLC.

**Methods:**

A systematic literature search of 7 electronic databases was conducted for randomized, placebo-controlled trials enrolling patients diagnosed with LS-SCLC who received chemoradiotherapy combined with immunotherapy. Trials with unstratified LS-SCLC samples were excluded. A subsequent meta-analysis was performed. This study was registered with the International Prospective Register of Systematic Reviews (PROSPERO) under accession number CRD42024607159.

**Results:**

Nine trials comprising 1,853 patients were included. CCRT combined with immunotherapy exhibited a significant favorable trend in survival outcomes, with hazard ratios (HRs) for median progression-free survival (mPFS) and median overall survival (mOS) of *0.838* (95% confidence interval [CI]: 0.742–0.947) and 0.847 (95% CI: 0.736–0.975), especially in the consolidation group with HRs for mPFS and mOS of 0.793 (95% CI: 0.675–0.930) and 0.757 (95% CI: 0.551–1.040), respectively.

**Conclusion:**

CCRT combined with immunotherapy improved survival in patients with LS-SCLC, especially in the consolidation immunotherapy group. The treatment regimen was generally well tolerated, with an acceptable incidence of treatment-related adverse events (TRAEs).

**Systematic review registration:**

https://www.crd.york.ac.uk/prospero/, identifier CRD42024607159.

## 1 Introduction

SCLC represents one of the most aggressive forms of lung cancer, characterized by rapid progression and a high propensity for metastasis. SCLC accounts for approximately 15% of all lung cancer diagnoses, with one-third of patients presenting with limited-stage (LS) disease at the time of diagnosis, leading to a dismal five-year survival rate of approximately 10% to 30% (1–3).The clinical landscape for SCLC is marked by significant challenges, as traditional treatment modalities, including chemotherapy and radiotherapy, often yield only transient responses and are associated with substantial toxicities. The limitations of current therapeutic strategies underscore the urgent need for innovative approaches that improve efficacy while minimizing toxicities.

Current therapeutic approaches primarily focus on cCRT, with regimens such as etoposide and platinum-based drugs serving as the backbone of LS-SCLC management. However, the development of chemoresistance poses a significant challenge, with many patients experiencing disease relapse shortly after initial therapy (4, 5). Moreover, the lack of targeted therapies and the limited understanding of the tumor microenvironment complicate treatment efforts(1) further, resulting in a pressing need for comprehensive research that addresses these deficiencies.

Despite the fact that numerous clinical trials of chemoimmunotherapy for extensive-stage small-cell lung cancer(ES-SCLC) have yielded positive results—including IMpower133 (6), CASPIAN (7), CAPSTONE-1 (8), ASTRUM-005 (9), and RATIONALE-312 (10) —immunotherapy has continued to achieve breakthroughs in improving survival outcomes for ES-SCLC patients. However, immunotherapy had not yielded significant breakthroughs in LS-SCLC until the recent ADRIATIC study (11), which demonstrated that durvalumab administered after chemoradiotherapy exerts a significant antitumor effect with respect to PFS and OS. However, the outcomes of tremelimumab in this trial have not yet been published, and there is still a lack of robust evidence to support the efficacy of other immune checkpoint inhibitors (ICIs). Whether chemoradiotherapy combined with immunotherapy has a widely applicable positive synergistic effect in LS-SCLC similar to ES-SCLC highlights the necessity for a systematic review and meta-analysis to synthesize existing data, clarify the efficacy of combined treatment approaches, and identify patient subgroups that may benefit most from such strategies.

Therefore, our primary objective was to evaluate the efficacy and safety of combined chemoradiotherapy and immunotherapy in patients with LS-SCLC, specifically focusing on mPFS and mOS as primary endpoints. Additionally, we aimed to assess the objective response rate (ORR) and the incidence of TRAEs, and to perform subgroup analyses. By employing rigorous methodologies in line with PRISMA guidelines and synthesizing data from multiple randomized controlled trials (RCTs), this study aspires to provide valuable insights that could influence clinical practice and enhance patient outcomes in LS-SCLC management. Ultimately, this research aims to provide comprehensive evidence to clinicians and patients in making informed treatment decisions.

## 2 Materials and methods

### 2.1 Literature search

The present investigation adhered to the PRISMA guidelines. To ensure comprehensiveness, the following Medical Subject Headings (MeSH) terms were used: “immunotherapy”, “chemotherapy”, “radiotherapy”, and “small cell lung cancer”. Relevant studies were searched in multiple databases, including PubMed, Embase, Web of Science, the Cochrane Library, China National Knowledge Infrastructure (CNKI), and 2 other Chinese databases(China Science and Technology Journal Database, Wanfang). The literature search was independently conducted by 2 authors and included all studies published up to January 2026. This study was registered with the International Prospective Register of Systematic Reviews(PROSPERO) under accession number CRD42024607159.

### 2.2 Inclusion criteria

The inclusion and exclusion criteria were as follows (1): RCTs enrolling patients with pathologically confirmed LS-SCLC who received combined chemoradiotherapy and immunotherapy; (2) Patients who had undergone surgical resection were excluded. Studies involving irrelevant or non-specific populations, and those that reported insufficient or duplicate data were excluded. If results from the same RCT were published in multiple journals, the most comprehensive or recent publication was selected to ensure inclusion of the most up-to-date data.

### 2.3 Data extraction

Two independent authors screened the literature records, carefully evaluated the full-text articles, and extracted relevant data from eligible studies, including publication year, national clinical trial registration number, study authors, therapeutic agents, study design, comparator, study region, age range, and treatment duration. Publication bias was assessed using funnel plots.

### 2.4 Flow diagram

**Figure 1:**
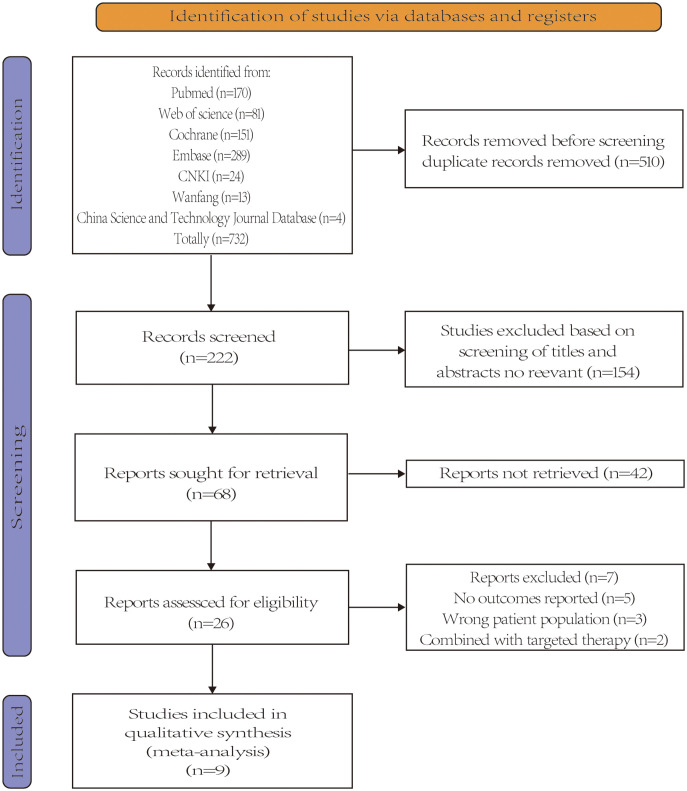
Flow diagram of meta-analysis.

The study selection and meta-analysis process is shown in **Figure 1**.

### 2.5 Quality assessment

Data were extracted in accordance with the predefined inclusion and exclusion criteria. Duplicate studies and ineligible studies were excluded, and the relevant data were compiled into a standardized database. Two authors extracted the data independently, and a third author cross-checked the data to resolve any inconsistencies. Data consistency was confirmed via statistical analysis.

### 2.6 Data synthesis

The primary outcomes of interest included mPFS, defined as the time from treatment initiation to the first documentation of disease progression or all-cause death (whichever occurred first), and mOS, defined as the time from randomization to all-cause death. Secondary outcomes included ORR (a short-term efficacy evaluation indicator), the incidence of TRAEs (safety assessment indicators related to treatment), and subgroup analyses of immunotherapy sequencing relative to cCRT, radiotherapy fractionation regimens, types of ICIs and whether prophylactic cranial irradiation (PCI) was used.

### 2.7 Statistical analysis

HRs with corresponding 95% CIs for each outcome across the included trials were pooled and presented in forest plots. Cochran’s Q test and I² statistic were used to assess statistical heterogeneity; a fixed-effects model (inverse variance method) was used if I² < 50% or P > 0.05, whereas a random-effects model (DerSimonian-Laird method) was applied otherwise. To assess the robustness of the study results, a one-study-at-a-time sensitivity analysis was performed to evaluate the impact of individual studies on the overall pooled outcomes. Statistical analyses were performed using Stata Release 18.0 (StataCorp LLC, College Station, TX, USA) and Python 3.12 (Python Software Foundation, Wilmington, DE, USA).

## 3 Results

### 3.1 Characteristics of the studies

The initial literature search retrieved 732 studies. After removal of duplicates and review of the full texts, 9 studies comprising 1,853 participants fulfilled the inclusion criteria and were included (11–19). All included studies administered chemoradiotherapy combined with at least 1 type of ICIs. Among the included studies, 5 trials (11–15) focused on consolidation immunotherapy administered after cCRT, 3 studies (16, 17, 19) (encompassing a total of 4 cohorts) employed cCRT concurrently combined with immunotherapy followed by consolidation immunotherapy, and 1 study (18) exclusively used induction immunotherapy combined with consolidation immunotherapy. Regarding radiotherapy fractionation regimens, 2 studies used conventional fractionation, 3 studies used hyperfractionation, and 1 study used hypofractionation. The remaining studies applied a combination of hyperfractionated and conventional radiotherapy. For immunotherapeutic agents, 4 studies administered PD-L1 inhibitor monotherapy, whereas the other 5 studies (with a total of 6 cohorts) used PD-1-based immune checkpoint inhibitors. Among these 5 studies, 4 datasets involved PD-1 inhibitor monotherapy, and the other 2 involved combination immunotherapies (anti-CTLA4 and anti-TIGIT). Baseline characteristics of the eligible studies are presented in **Table 1**.

**Table 1:** Baseline characteristics of studies included in the systematic review and meta-analysis.

NCT	Study	Publication year	Sample size	Timing of immunotherapy	Radiotherapy fractionation regimen	Radiation dose	Drugs	ICIs
NCT04418648	P. Zhang	2025	96	Consolidation	Hyperfractionation	45Gy/30f(Bid) or 60-65Gy/24-26f(Bid)	Toripalimab	PD-1
NCT03703297	Spigel, D.	2024	530	Consolidation	Hyperfractionation + Conventional Fractionation	45Gy/30f(Bid) or 60-66Gy(Qd)	Durvalumab	PD-L1
NCT03540420	Bjorn Henning Gronberg	2025	170	Consolidation	Hyperfractionation	45Gy/30f(Bid) or 60Gy/40f	Atezolizumab	PD-L1
NCT05443646	Yin Yang	2025	144	Consolidation	Hypofractionation	45Gy/15f	Serplulimab	PD-1
NCT02046733	Peters, S	2022	153	Consolidation	Hyperfractionation + Conventional Fractionation	NA	Nivolumab + Ipilimumab	PD-1 + anti CTLA-4
NCT04952597	Youling Gong	2025	85	Concurrent + Consolidation	Conventional Fractionation	60-70Gy(Qd)	Tislelizumab	PD-1
NCT04952597	Youling Gong	2025	84	Concurrent + Consolidation	Conventional Fractionation	60-70Gy(Qd)	Ociperlimab + Tislelizumab	Anti-TIGIT + PD-1
NCT03585998	Sehhoon Park	2022	50	Concurrent + Consolidation	Conventional Fractionation	52.5Gy/25f(Qd)	Durvalumab	PD-L1
NCT03811002	K. Higgins	2026	544	Concurrent + Consolidation	Hyperfractionation + Conventional Fractionation	45Gy/30f(Bid) or 66Gy/33f(Qd)	Atezolizumab	PD-L1
ChiCTR2000032275	D. Liu	2025	40	Induction + Consolidation	Hyperfractionation	45Gy(Bid)	Camrelizumab	PD-1

### 3.2 PFS evaluation

**Figure 2:**
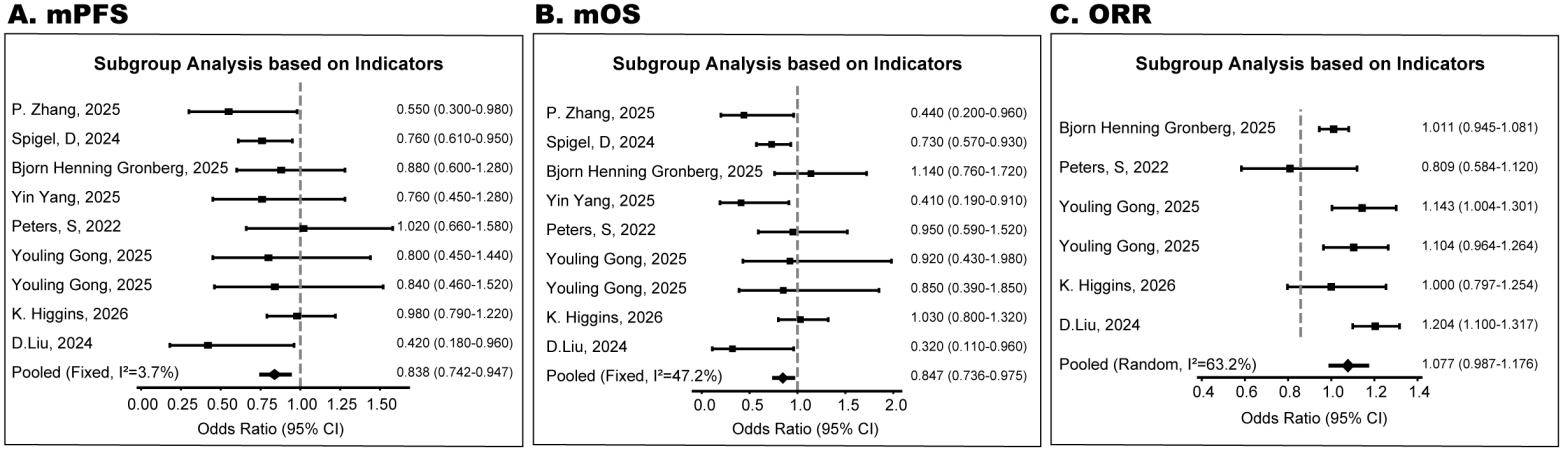
Forest plots for the efficacy of immunotherapy on mPFS **(A)**, mOS **(B)**, and ORR **(C)** across individual studies in LS-SCLC patients who received chemoradiotherapy.

Assessment of PFS was divided into 2 components: mPFS and annual PFS rate. For the overall mPFS, a fixed-effects model was used for subsequent analysis due to the low statistical heterogeneity(I² = 3.7%). Immunotherapy combined with cCRT reduced the overall risk to 0.838 (95% CI: 0.742–0.947) (**Figure 2A**). Sensitivity analysis revealed that the conclusion was not affected by the exclusion of any study (**Table 2**). The HRs of 1-year and 2-year PFS rates were higher in the group receiving combined cCRT and immunotherapy compared with the non-immunotherapy group, indicating a higher risk of disease progression or death (**Supplementary Figure 1**).

**Table 2:** Sensitivity analysis for mPFS.

Model	Estimate	95% Conf. interval
Adjusted model 1	0.87	[0.77, 0.98]
Adjusted model 2	0.89	[0.77, 1.04]
Adjusted model 3	0.85	[0.74, 0.97]
Adjusted model 4	0.86	[0.75, 0.97]
Adjusted model 5	0.84	[0.74, 0.95]
Adjusted model 6	0.85	[0.75, 0.97]
Adjusted model 7	0.85	[0.75, 0.97]
Adjusted model 8	0.80	[0.69, 0.92]

Adjusted model 1: excluding the study by P. Zhang; Adjusted model 2: excluding the study by Spigel, D; Adjusted model 3: excluding the study by Bjorn Henning Gronberg; Adjusted model 4: excluding the study by Yin Yang; Adjusted model 5: excluding the study by Peters, S; Adjusted model 6: excluding the study by Youling Gong; Adjusted model 7: excluding the study by Youling Gong; Adjusted model 8: excluding the study by K. Higgins.

### 3.3 OS evaluation

Assessment of OS was also divided into 2 components: mOS and annual OS rate. For the overall mOS, combined cCRT and immunotherapy reduced the overall risk of all-cause death to 0.847 (95% CI: 0.736–0.975) (**Figure 2B**). Results of the sensitivity analysis indicated that the conclusion was not driven by any single study alone, but 2 studies—Spigel (2024) and Higgins (2026)—exerted a substantial impact on the magnitude and statistical significance of the results (**Table 3**). As for the annual OS rate, the I² values for 1-year, 2-year, and 3-year OS rates were 25.7%, 70.7%, and 13.6%, respectively. Compared with the non-immunotherapy group, the 3-year OS rate was significantly increased (**Supplementary Figure 2**).

**Table 3:** Sensitivity analysis for mOS.

Model	Estimate	95% Conf. interval
Adjusted model 1	0.87	[0.75, 1.00]
Adjusted model 2	0.91	[0.77, 1.08]
Adjusted model 3	0.81	[0.70, 0.95]
Adjusted model 4	0.87	[0.75, 1.00]
Adjusted model 5	0.84	[0.72, 0.97]
Adjusted model 6	0.84	[0.73, 0.97]
Adjusted model 7	0.85	[0.73, 0.98]
Adjusted model 8	0.77	[0.65, 0.92]
Adjusted model 9	0.86	[0.75, 0.99]

Adjusted model 1: excluding the study by P. Zhang; Adjusted model 2: excluding the study by Spigel, D; Adjusted model 3: excluding the study by Bjorn Henning Gronberg; Adjusted model 4: excluding the study by Yin Yang; Adjusted model 5: excluding the study by Peters, S; Adjusted model 6: excluding the study by Youling Gong; Adjusted model 7: excluding the study by Youling Gong; Adjusted model 8: excluding the study by K. Higgins; Adjusted model 9: excluding the study by D.Liu.

### 3.4 ORR evaluation

A pooled HR for ORR was 1.077 (95% CI: 0.987–1.176) (**Figure 2C**). We performed a leave-one-out sensitivity analysis to evaluate the robustness of this results (**Table 4**). The pooled HRs for the remaining studies ranged from 1.04 (95% CI: 0.99–1.09) after excluding D. Liu (2024), and more importantly, the 95% CI crossed the line of no effect (95% CI: 0.99–1.09), rendering the result statistically non-significant. In contrast, the exclusion of any other study maintained both the magnitude and statistical significance of the pooled estimate.

**Table 4:** Sensitivity analysis for ORR.

Model	Estimate	95% Conf. interval
Adjusted model 1	1.14	[1.07, 1.21]
Adjusted model 2	1.08	[1.04, 1.13]
Adjusted model 3	1.07	[1.02, 1.12]
Adjusted model 4	1.07	[1.02, 1.13]
Adjusted model 5	1.08	[1.03, 1.13]
Adjusted model 6	1.04	[0.99, 1.09]

Adjusted model 1: excluding the study by Bjorn Henning Gronberg; Adjusted model 2: excluding the study by Peters, S; Adjusted model 3: excluding the study by Youling Gong; Adjusted model 4: excluding the study by Youling Gong; Adjusted model 5: excluding the study by K. Higgins; Adjusted model 6: excluding the study by D.Liu.

### 3.5 Subgroup analyses

#### 3.5.1 Impact of the timing of immunotherapy administration

**Figure 3:**
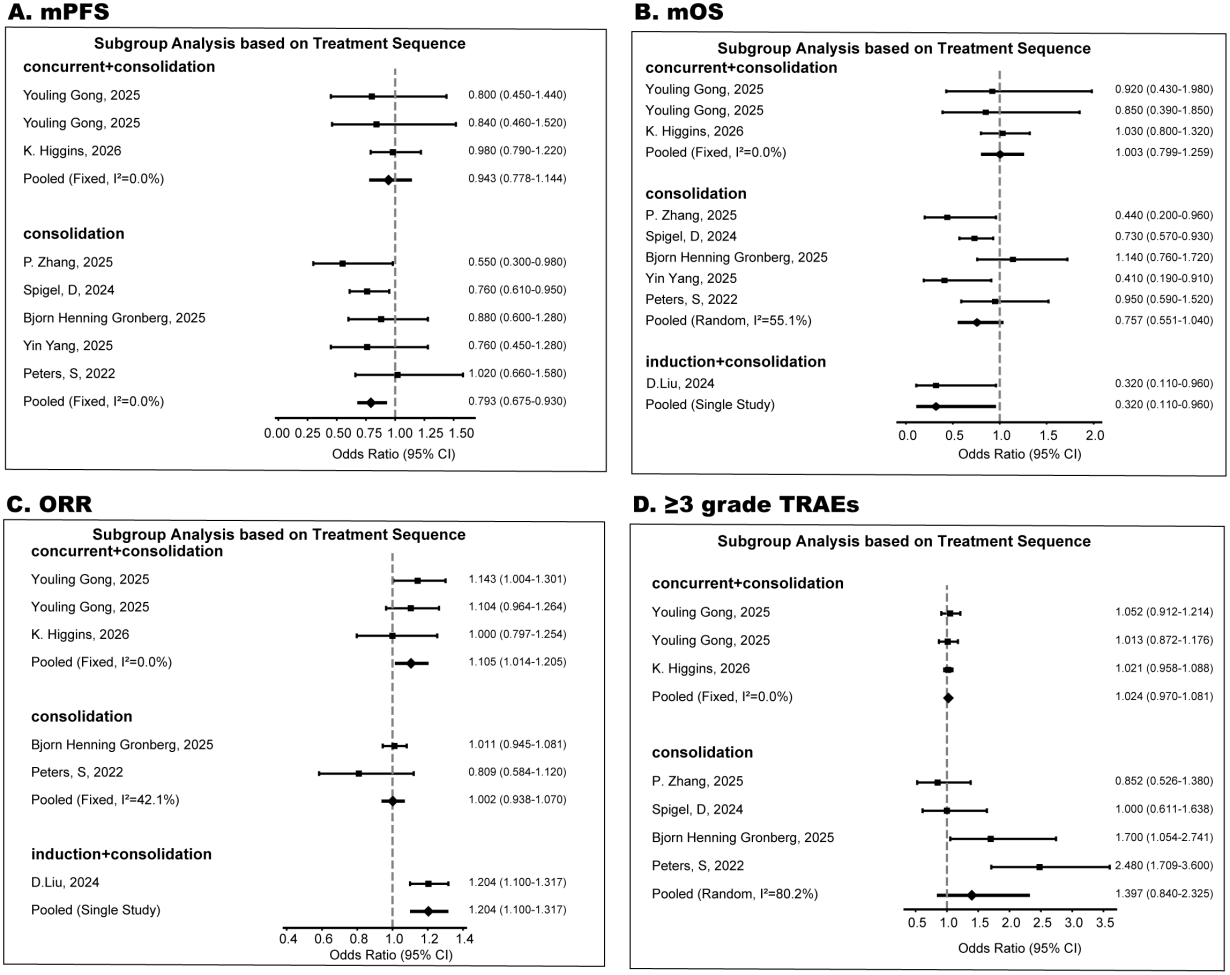
Forest plots for subgroup analyses by different immunotherapy regimens. **(A)** compare mPFS between concurrent consolidation immunotherapy and consolidation-only immunotherapy subgroups; **(B, C)** display pooled effects on mOS and ORR across concurrent consolidation immunotherapy, consolidation-only immunotherapy, and induction plus consolidation immunotherapy, while **(D)** contrasts the incidence of ≥3 grade TRAEs between concurrent consolidation immunotherapy and consolidation-only immunotherapy subgroups.

For mPFS, the HRs were 0.793 (95% CI: 0.675–0.930) in the consolidation group and 0.943 (95% CI: 0.778–1.144) in the concurrent+consolidation group (**Figure 3A**). For mOS, the HR was 0.757 (95% CI: 0.551–1.040) in the consolidation group, whereas no significant risk of death reduction was noted in the concurrent+consolidation group (HR = 1.003, 95% CI: 0.799–1.259) (**Figure 3B**). As for ORR, an HR of 1.002 (95% CI: 0.938–1.070) in the consolidation subgroup showed a better short-term efficacy than the concurrent+consolidation subgroup (HR = 1.105, 95% CI: 1.014–1.205) (**Figure 3C**). We further compared AEs between the immunotherapy consolidation group and the concurrent+consolidation group (the induction+consolidation group was excluded because no AEs data were reported). For grade ≥3 TRAEs, the pooled HR was 1.397 (95% CI: 0.840–2.325) in the consolidation group and 1.024 (95% CI: 0.970–1.081) in the concurrent+consolidation group (**Figure 3D**). Since all available data on immune-related adverse events (irAEs) were derived from the concurrent +consolidation group, comparison of irAEs between the 2 groups was not feasible.

#### 3.5.2 Impact of PD-1 and PD-L1 inhibitors

**Figure 4:**
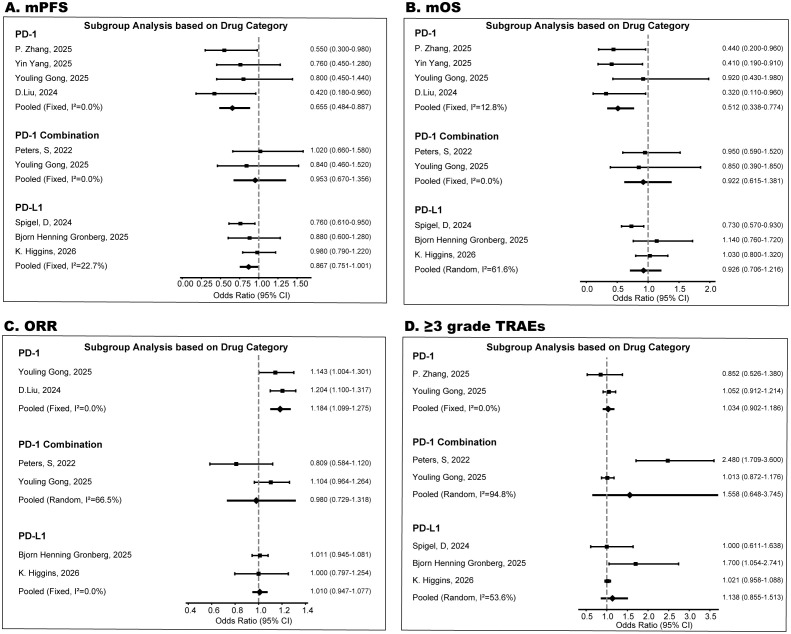
Forest plots illustrating the effects on mPFS **(A)**, mOS **(B)**, ORR **(C)**, and ≥3 grade TRAEs **(D)** for LS-SCLC patients treated with anti-PD-1 monotherapy, PD-1-based combination therapy, or anti-PD-L1 monotherapy.

7 studies were used to perform subgroup analyses for mPFS and mOS. The HRs for the PD-1 and PD-L1 subgroups were 0.655 (95% CI: 0.484–0.887) and 0.867 (95% CI: 0.751–1.001) in mPFS, respectively (**Figure 4A**). For mOS, the HRs for these 2 subgroups were 0.512 (95% CI: 0.338–0.774) and 0.926 (95% CI: 0.706–1.216) (**Figure 4B**). ORR was analyzed across 4 trials, with corresponding HRs of 1.184 (95% CI: 1.099–1.275) and 1.010 (95% CI: 0.947–1.077) for the PD-1 and PD-L1 subgroups, respectively (**Figure 4C**). Grade ≥3 TRAEs was analyzed across 5 trials, with corresponding HRs of 1.034 (95% CI: 0.902–1.186) and 1.138 (95% CI: 0.855–1.513) for the PD-1 and PD-L1 subgroups, respectively (**Figure 4D**).

To further compare the roles of PD-1 and PD-L1 inhibitors in different timing of immunotherapy administration, we evaluated their effects on mPFS, mOS, ORR, and grade ≥3 TRAEs in the consolidation subgroup and the concurrent+consolidation subgroup, respectively. In the consolidation subgroup, the PD-1 inhibitor group was associated with lower HRs both in the mPFS (**Supplementary Figure 3A**) and the mOS (**Supplementary Figure 3B**) compared with the PD-L1 inhibitor group (mPFS: 0.660 vs. 0.789; mOS: 0.425 vs. 0.884). For grade ≥3 TRAEs, the HR was 0.852 in the PD-1 group, while a trend toward higher AEs risk in the PD-L1 group (**Supplementary Figure 3C**). In the concurrent+consolidation subgroup, the PD-1 inhibitor group showed a marked reduction in HR for mPFS and mOS compared with the PD-L1 group (mPFS: 0.800 vs. 0.980; mOS: 0.920 vs. 1.030) (**Supplementary Figures 5A, B**), while an increase in HR for grade ≥3 TRAEs (1.052 vs. 1.021) (**Supplementary Figure 5D**). Furthermore, no significant improvement in ORR was observed between the PD-L1 inhibitor group and the PD-1 inhibitor group in the concurrent+consolidation subgroup (**Supplementary Figure 5C**). No further analysis was performed for ORR in the consolidation subgroup due to insufficient available data.

#### 3.5.3 Comparison of monotherapy and combination therapy

A comparison between monotherapy and combination therapy was performed between PD-1 inhibitor monotherapy and dual-agent immunotherapy with respect to mPFS, mOS and ORR. Totally, 2 datasets were assigned to the PD-1 combination group, 1 combining with the anti-CTLA-4 inhibitor in the consolidation subgroup (15), while the other combined with an anti-TIGIT inhibitor in the concurrent+consolidation subgroup (19). Both HRs for mPFS and mOS were significantly decreased in the PD-1 inhibitor monotherapy group (mPFS: 0.655 vs. 0.953; mOS: 0.512 vs. 0.922), compared with the PD-1-based combination immunotherapy subgroup (**Figures 4A, B**). As for ORR, the HR of PD-1 inhibitor monotherapy was 1.184 (95% CI: 1.099–1.275), whereas PD-1-based combination immunotherapy was 0.980 (95% CI: 0.729–1.318) (**Figure 4C**). Furthermore, the HR for grade ≥ 3 TRAEs between 2 groups were 1.034 and 1.558 (**Figure 4D**).

We also performed further analyses between PD-1 and PD-1-based combination. The PD-1 group exhibited significantly reduced HRs compared with the PD-1 combination group (mPFS: 0.660 vs. 1.020; mOS: 0.425 vs. 0.950; ≥ 3 grade TRAEs: 0.852 vs. 2.480) in the consolidation subgroup (**Supplementary Figure 3**). In contrast, the PD-1 group showed increased HRs in the concurrent+consolidation subgroup (mOS: 0.920 vs. 0.850; ≥3 grade TRAEs: 1.052 vs. 1.013), except for mPFS (0.800 vs. 0.840) (**Supplementary Figure 5**).

In the consolidation group, the sensitivity analysis showed that the findings for mPFS, mOS and ≥3 grade TRAEs were robust, but the mOS effect was not statistically significant (**Supplementary Figure 4**). In the concurrent+consolidation group, the overall conclusion was relatively robust, but the study by K. Higgins had a significant impact on the pooled effect (**Supplementary Figure 6**).

#### 3.5.4 Impact of radiotherapy fractionation regimen

**Figure 5:**
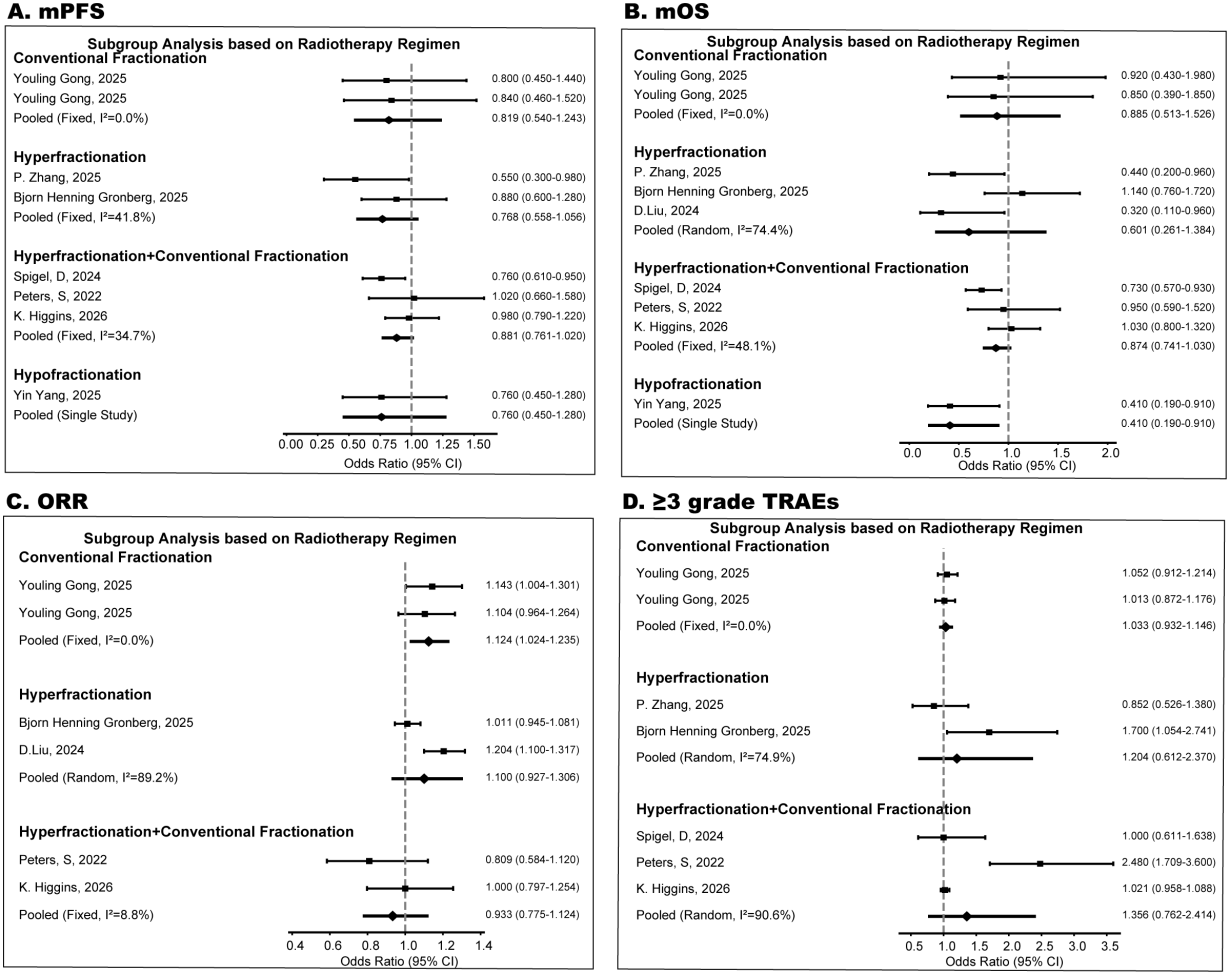
Forest plots comparing the effects of different radiotherapy fractionation subgroups. **(A, B)** compare mPFS and mOS among conventional fractionation, hyperfractionation, combined hyperfractionation plus conventional fractionation, and hypofractionation; **(C, D)** contrast ORR and ≥3 grade TRAEs across conventional fractionation, hyperfractionation, and combined hyperfractionation plus conventional fractionation radiotherapy regimens.

Among the subgroups of hyperfractionation, conventional fractionation and hyperfractionation+conventional fractionation, the HR values of mPFS were 0.768, 0.819 and 0.881, respectively; the values of mOS were 0.601, 0.885 and 0.874, respectively. The hypofractionation subgroup had the lowest HR values, with 0.760 for mPFS and 0.410 for mOS (**Figures 5A, B**). Regarding ORR, the HRs gradually increased across the hyperfractionation+conventional fractionation, hyperfractionation, and conventional fractionation subgroups in sequence (0.933 vs. 1.100 vs. 1.124; **Figure 5C**), whereas an opposite trend was observed in ≥3 grade TRAEs (1.356 vs. 1.204 vs. 1.033; **Figure 5D**).

#### 3.5.5 Impact of PCI

We included only 3 studies that reported data on PCI (11, 13, 17), with a random-effects model applied accordingly to the high heterogeneity (I² = 88.0%). The pooled HR was 1.261 (95% CI: 0.778–2.044), with no statistical significance (**Supplementary Figure 7**).

### 3.6 Safety analyses

**Figure 6:**
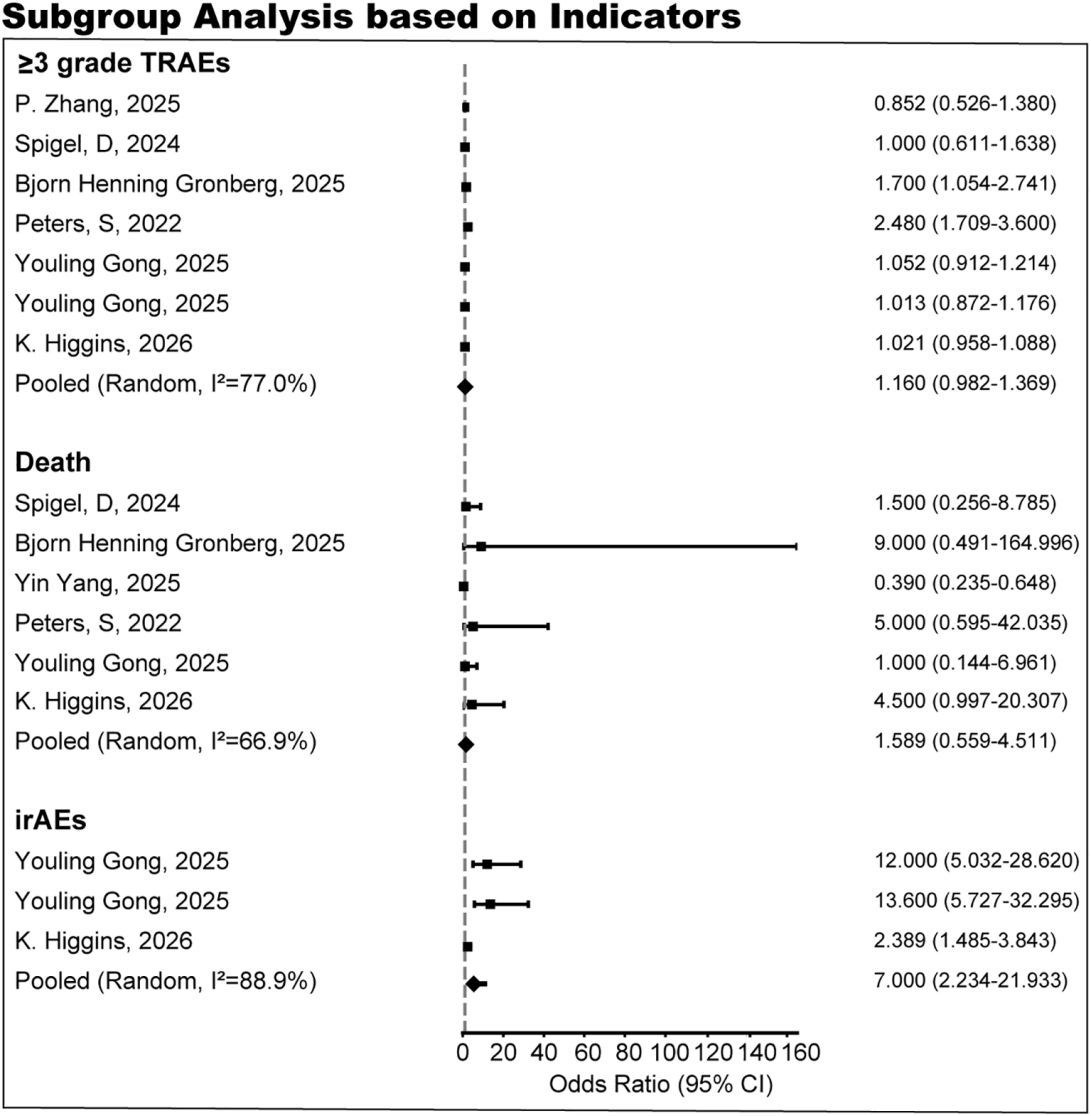
Forest plot for studies with ≥3 grade TRAEs, death, and irAEs in LS-SCLC patients who received immunotherapy combined with chemoradiotherapy.

Safety analyses were performed from 6 studies (7 cohorts) reporting AEs. The pooled HR for grade ≥3 TRAEs in the immunotherapy group was 1.160 (95% CI: 0.982–1.369), and that for treatment-related mortality was 1.589 (95% CI: 0.559–4.511). Among the only 2 studies reporting irAEs, the pooled HR was 7.000 (95% CI: 2.234–21.933). No statistically significant differences were observed in any high-grade AEs, with the exception of irAEs (**Figure 6**). Furthermore, significant differences also observed in the low-grade AEs summarized in **Table 5** (except anemia). This sensitivity analysis indicates that the overall conclusion of “no statistically significant difference” is not robust, which is entirely dependent on the inclusion of the study by K. Higgins (2026). When this study was omitted, the pooled effect became statistically significant (HR = 1.10, 95% CI: 1.01–1.21), suggesting a potential harmful effect. In contrast, the exclusion of any other individual study consistently yielded non-significant results (**Table 6**).

**Table 5:** The summary results for TRAEs.

Outcomes	Samples	RR	95% CI	Heterogeneity	Z-test
anemia	2	1.013	0.922, 1.112	I^2^ = 0.0%, P = 0.796	P=0.789
Nausea	2	1.209	1.067, 1.369	I^2^ = 0.0%, P = 0.617	P=0.003
leukopenia	2	1.222	1.073, 1.393	I^2^ = 0.0%, P = 0.844	P=0.003
Pneumonitis	4	1.428	1.091, 1.869	I^2^ = 9.2%, P = 0.347	P=0.009
discontinuation	4	3.496	1.766, 6.921	I^2^ = 60.0%, P = 0.057	P<0.001

**Table 6:** Sensitivity analysis for ≥3 TRAEs.

Model	Estimate	95% Conf. interval
Adjusted model 1	1.05	[0.99, 1.11]
Adjusted model 2	1.05	[0.99, 1.10]
Adjusted model 3	1.04	[0.99, 1.10]
Adjusted model 4	1.03	[0.97, 1.08]
Adjusted model 5	1.05	[0.99, 1.11]
Adjusted model 6	1.05	[0.99, 1.11]
Adjusted model 7	1.10	[1.01, 1.21]

Adjusted model 1: excluding the study by P. Zhang; Adjusted model 2: excluding the study by Spigel, D; Adjusted model 3: excluding the study by Bjorn Henning Gronberg; Adjusted model 4: excluding the study by Peters, S; Adjusted model 5: excluding the study by Youling Gong; Adjusted model 6: excluding the study by Youling Gong; Adjusted model 7: excluding the study by K. Higgins.

### 3.7 Bias assessments

**Figure 7:**
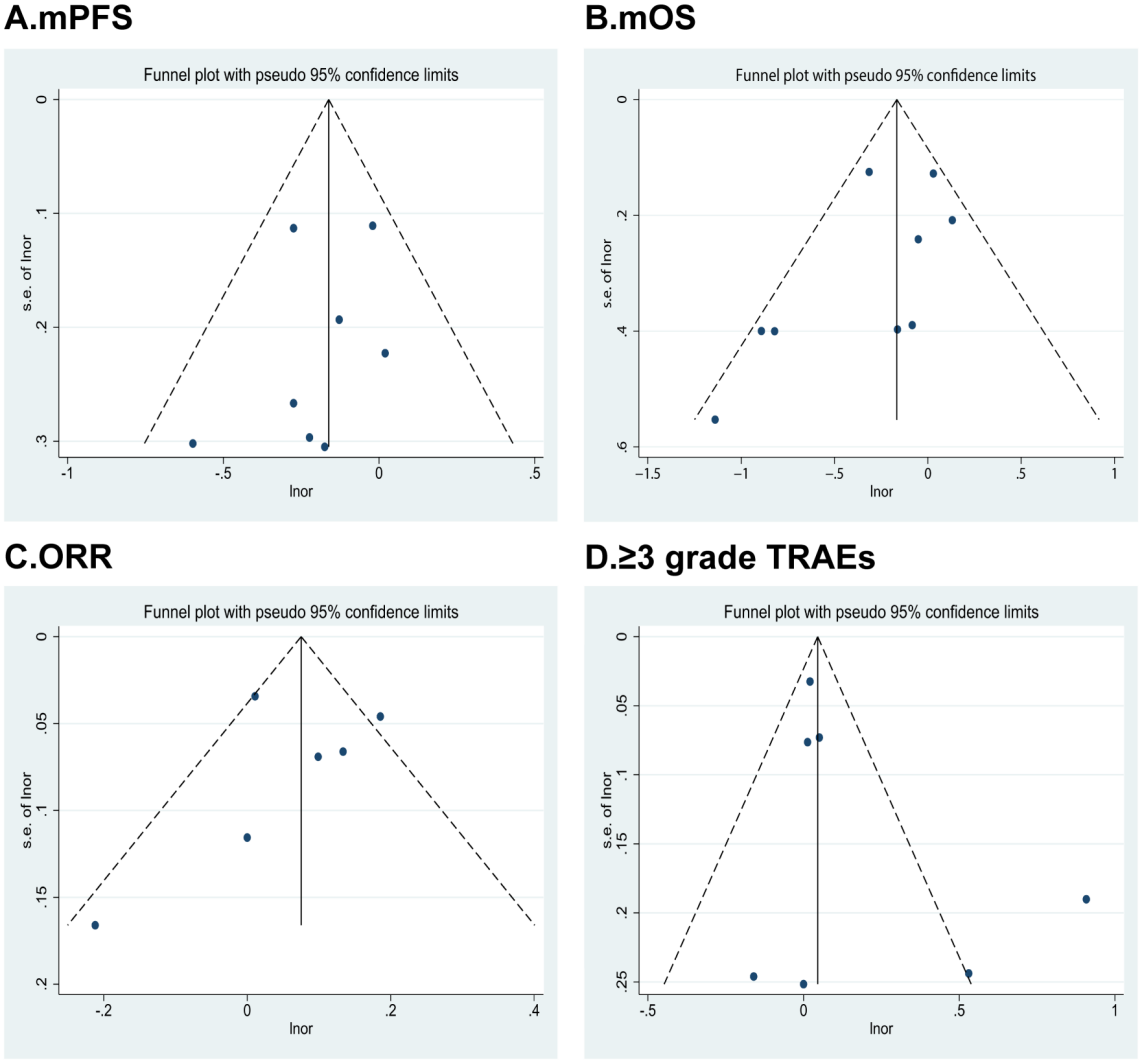
Funnel plots for publication bias assessment of mPFS **(A)**, mOS **(B)**, ORR **(C)**, and ≥3 grade TRAEs **(D)**.

Bias assessments are summarized in **Figure 7**. Publication bias was assessed using funnel plots, where the centrally symmetric funnel-shaped region represents the 95% CI. In the present study, funnel plot for mOS appeared relatively symmetrical, indicating no obvious evidence of publication bias. The funnel plots for mPFS and ORR showed an overall asymmetric distribution, suggesting potential small-study effects or publication bias. The funnel plot for grade ≥3 TRAEs demonstrated noticeable asymmetry, indicating potential publication bias or selective reporting of AEs.

## 4 Discussion

LS-SCLC is a highly malignant type of lung cancer that poses a significant threat to patients’ lives. Despite the current standard treatment of cCRT for LS-SCLC, the prognosis remains unsatisfactory. Hence, it is essential to explore more effective treatment options for LS-SCLC.

Our systematic review and meta-analysis was based on 9 eligible studies involving 1,853 patients to evaluate the safety and efficacy of chemoradiotherapy combined with immunotherapy in the treatment of LS-SCLC. This research provides valuable insights into the potential benefits of the combined treatment approach.

Our overall analysis revealed that the combination of chemoradiotherapy and immunotherapy significantly improves mPFS and mOS in LS-SCLC, with HR of 0.838 (95% CI: 0.742–0.947) for mPFS and 0.847 (95% CI: 0.736–0.975) for mOS. These findings underscore the synergistic effect of dual therapy, suggesting a paradigm shift in treatment strategies for LS-SCLC, where historically the prognosis has been poor. Analysis of TRAEs incidence demonstrated that the addition of immunotherapy was associated with increased toxicity, which warrants clinical attention but remains manageable, thus reinforcing the feasibility of implementing this regimen in clinical practice.

Both the 2025 CSCO guidelines and the NCCN guidelines list durvalumab as a Category 1 recommended standard treatment for consolidation therapy after cCRT in patients with LS. Our analysis further supports the conclusion that immunotherapy combined with cCRT demonstrates a trend toward survival benefit in patients with LS-SCLC with a manageable safety profile, but this depends on the timing of immunotherapy intervention (20).

Mechanistically, radiotherapy can induce additional neoantigens, activate T-cells in the tumor draining lymph nodes (TDLNs) and increase the number of tumor infiltrating leukocytes (21–23). Therefore, it would be logical to predict that the administration of radiotherapy slightly before or at the same time as immune checkpoint blockade (ICB) would be a prerequisite to establishing the ideal tumor microenvironment (TME) (24). A preclinical study using a murine model demonstrated that consolidation immunotherapy improved both local and abscopal effects (25), which was confirmed in our further subgroup analyses that consolidation immunotherapy represents the optimal timing for immunotherapy administration in combination with cCRT which provides a longer survival benefit despite the greater high-grade treatment-related toxicity.

Nevertheless, discrepancies existed between mPFS and PFS rate, as well as between mOS and OS rate, which may be explained by the following factors: (1) Immunotherapy shows a time-dependent effect with delayed onset, resulting in even lower short-term survival due to toxicity or slow efficacy. This may also account for the negative result observed in ORR, a metric for assessing short-term efficacy. (2) Median survival based on HR has higher statistical power and reflects the overall trend, whereas annual survival is easily affected by early events and censoring. (3) HR and separated survival curves represent the gold standard for evaluating immunotherapy, and prolonged median survival with combination therapy is clinically meaningful (26). This indicates that the results of mPFS and mOS are more representative.

Additionally not all ICIs demonstrated comparable efficacy in consolidation therapy in LS-SCLC in the real-world (27, 28). The negative result of both PFS and OS in consolidation therapy with atezolizumab found by Bjørn H. Grønberg et al (13) also illustrates the importance of selecting the type of ICIs. Meanwhile, the NRG/Alliance LU005 trial, which represents the largest known trial of immunotherapy combined with cCRT in LS-SCLC showed no benefit from concurrent+consolidation immunotherapy equally (17). PD-L1 inhibitors demonstrate more consistent survival benefits in combination therapy in ES-SCLC, particularly establishing a new standard in first-line treatment (7–10). In contrast, PD-1 inhibitors show clear advantages only in first-line treatment for PD-L1-high non-small-cell lung cancer (NSCLC) (29), but exhibit limited efficacy in SCLC (only in ASTRUM-005 (9) and RATIONALE-312 (10)). However, this is inconsistent with our research findings. All the PD-1 group demonstrated a significant risk reduction in mPFS and mOS compared with the PD-L1 group, and grade ≥3 TRAEs did not increase. This result was also observed in the better pathological complete response (pCR), major pathological response (MPR) and disease-free survival (DFS) in the perioperative immunotherapy of multiple solid tumors (30).

In our study, more than 2 immune inhibitors applied together would lower the survival efficacy, which is partially consistent with previous research findings (31–33). Dual-agent immunotherapy usually targets 2 different immune checkpoints. While this complementary effect amplifies the anti-tumor immune response, it may also lead to excessive immune activation against normal tissues, triggering severe irAEs (34). This conclusion is well supported by the CLOVER study that compared with the durvalumab monotherapy group, the durvalumab+tremelimumab combination group showed a marked increase in the incidence of TRAEs and a worse mPFS (35). Our findings also align with this conclusion that the PD-1 monotherapy group exhibited favorable survival benefits and a lower incidence of AEs.

Stratifying patients by biomarker status represents a key strategy for optimizing treatment outcomes. SCLC-A (ASCL1-high) is the most common subtype. Evidence suggests that the proportion of the SCLC-A subtype is indeed higher in LS-SCLC than in ES-SCLC (36). Additionally, Cluster of Differentiation 8 positive (CD8+) T cells, and highly expressed Major Histocompatibility Complex Class I (MHC-I) collectively contribute to a more active immune microenvironment in LS-SCLC (37, 38). Furthermore, Yin Yang et al. demonstrated that ctDNA could also effectively predict the efficacy of immunotherapy (14). Although preliminary exploration of immunotherapeutic efficacy biomarkers has existed since the ADRIATIC trial, additional large-scale RCTs are required.

In addition, we investigated the impact of radiotherapy fractionation regimens on immunotherapy efficacy. In our study, the hyperfractionation subgroup achieved the most favorable survival benefit, followed by the conventional fractionation and hyperfractionation+conventional. The hypofractionation subgroup showed the best survival outcomes. The RTOG 0538 study and long-term follow-up data from the CONVERT trial showed that 45 Gy/30 fractions bid, as the current standard radiotherapy regimen, achieved similar efficacy to 66 Gy or 70 Gy qd regimens, with comparable safety and improved treatment compliance (39, 40). Furthermore, hypofractionated radiotherapy was associated with shorter treatment duration and lower toxicity, while yielding comparable 2-year OS rates to conventional radiotherapy, and could be a promising alternative for LS-SCLC (41).

Immunotherapy can significantly reduce extrathoracic progression, particularly brain or central nervous system (CNS) metastases, and delay patient mortality (42, 43). However, our findings differ from those of the ADRIATIC trial, where PCI conferred significant survival benefits to patients (11). This discrepancy may be largely attributed to the substantial baseline bias among patients in the ADRIATIC study. In the era of immunotherapy, comparative data between PCI and regular magnetic resonance imaging (MRI) surveillance in patients with LS-SCLC remain scarce, which are warranted to guide clinical decision-making regarding the optimal choice.

Furthermore, our analysis revealed distinct safety profiles associated with these treatment modalities. Result from only 2 studies (17, 19) demonstrated irAEs, so we can’t distinguish the exact AEs results from immunotherapy and the effect of different ICIs on AEs is unknown. However, based on the available data, cCRT combined with immunotherapy did increase the incidence of lower-grade TRAEs, particularly in leukopenia and pneumonitis. This is correspond with the known theory established by preclinical studies that the application of ICIs with radiotherapy increases the incidence of lymphopenia and pneumonia (44, 45). Overall, the results regarding TRAEs were as we had anticipated and generally tolerable. However, due to the small amount of data, this combination should continue to be evaluated in more clinical trials.

The present study has several notable limitations that must be acknowledged to understand the reliability and applicability of the findings. Only 9 RCTs were included, which may restrict the stability of the pooled findings, especially there was merely 1 study investigating induction therapy. Although we may accept the presence of heterogeneity below 75%, there are still exist some relatively higher between-study heterogeneity such as those in TRAEs and ORR. This could be explained by the inclusion of several studies where the data themselves failed to reach statistical significance. Furthermore, in the sensitivity analysis, large-sample studies exerted an excessively strong influence and may dominate the final conclusions. Potential small-study effects observed in the funnel plot also warrant greater attention and cautious interpretation. Therefore, the optimal cCRT-immunotherapy regimen for LS-SCLC remains to be validated by additional high-quality clinical data, particularly large-scale phase III trials or well-designed real-world studies. Presently, the results of many clinical studies, such as NCT05623267 (46) and NCT04624204 (47), have not been fully published. Future research still needs more diverse populations and larger cohorts to provide guidance for the current clinical work.

Recently, antibody-drug conjugate (ADC) drugs, as a hot direction of new anti-cancer drugs, aim to block the immune inhibitory signaling of cancer cells, provide a new direction for immunotherapy in patients with LS-SCLC. For ES-SCLC, Ifinatamab Deruxtecan (I-DXd) has achieved a considerable ORR result, with remarkable median PFS and OS, particularly in patients with brain metastases due to its good blood-brain barrier penetration capability (48). Additionally, agents targeting specific antigens such as DLL3 (49), and SEZ6 (50) also show promising potential for the treatment of LS-SCLC.

## 5 Conclusion

In conclusion, as the first meta-analysis evaluating the role of cCRT combined with immunotherapy in LS-SCLC patients, our study suggests that cCRT combined with immunotherapy improves survival for patients with favorable outcomes in terms of mPFS and mOS and the treatment regimen was generally well-tolerated. Notably, our data analysis showed that consolidation immunotherapy, administered with either hyperfractionated or hypofractionated radiotherapy and PD-1 inhibitors was associated with greater survival benefits. Despite the high heterogeneity in some analyses, our research provides valuable insights into the treatment of LS-SCLC. Future studies should focus on long-term efficacy, potential resistance mechanisms, and the balance between efficacy and cost-effectiveness of immunotherapy.

## Data Availability

The original contributions presented in the study are included in the article/**Supplementary Material**. Further inquiries can be directed to the corresponding author.

## Glossary

cCRT: concurrent chemoradiotherapy
HR: hazard ratio
LS-SCLC: limited-stage small-cell lung cancer
AEs: adverse events
mPFS: median progression-free survival
mOS: median overall survival
CI: confidence interval
TRAEs: treatment-related adverse events
LS: limited-stage
ES-SCLC: extensive stage small-cell lung cancer
ICIs: immune checkpoint inhibitors
ORR: objective response rate
PRISMA: Preferred Reporting Items for Systematic Reviews and Meta-Analyses
RCTs: randomized controlled trials
MeSH: Medical Subject Headings
CNKI: China National Knowledge Infrastructure
PROSPERO: Prospective Register of Systematic Reviews
SE: standard error
PCI: prophylactic cranial irradiation
PFS: progression-free survival
OS: overall survival
PD-1: Programmed Cell Death Protein 1
PD-L1: Programmed Death-Ligand 1
irAEs: immune-related adverse events
SCLC: small-cell lung cancer
NSCLC: non-small-cell lung cancer
TDLNs: tumor draining lymph nodes
TME: tumor microenvironment
ICB: immune checkpoint blockade
pCR: pathological complete response
MPR: major pathological response
DFS: disease-free survival
ASCL1-high: SCLC-A
CD8+: Cluster of Differentiation 8 positive
MHC-I: Major Histocompatibility Complex Class I
CNS: central nervous system
MRI: magnetic resonance imaging
ADC: antibody-drug conjugate
I-DXd: Ifinatamab Deruxtecan.
