# Supplementary material for: Efficacy and safety of immunotherapy combined with chemoradiotherapy in limited-stage small cell lung cancer: a systematic evaluation and meta-analysis of randomized controlled trials
Source: Front Immunol. 2026 Jul 22;17:1731036. doi: 10.3389/fimmu.2026.1731036 (PMC13438153; doi:10.3389/fimmu.2026.1731036)
Supplement: Supplementary file 1 [file DataSheet1.pdf]

Fig. S1.

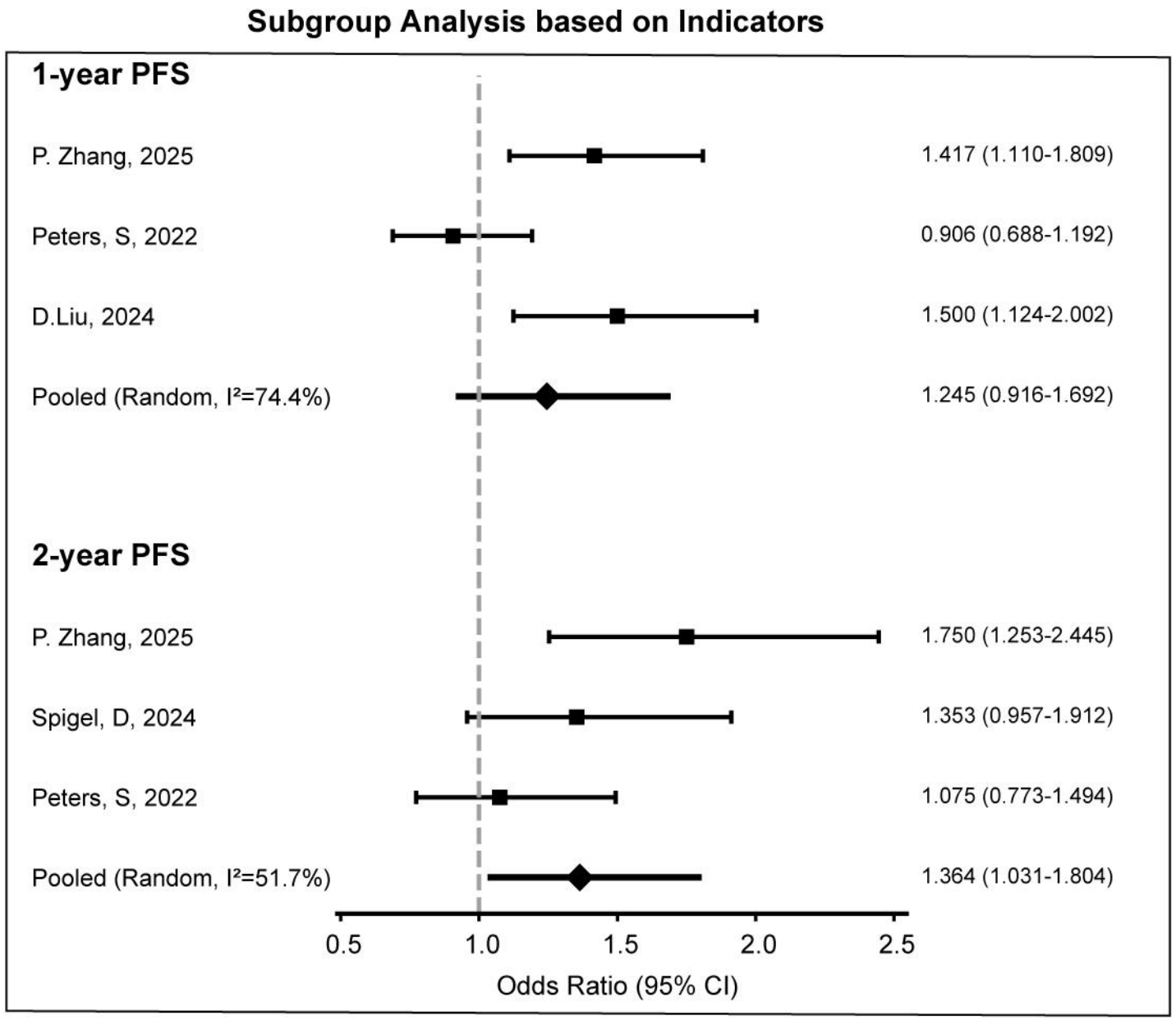

Fig. S1. 1-year and 2-year PFS rate.

Fig. S2.

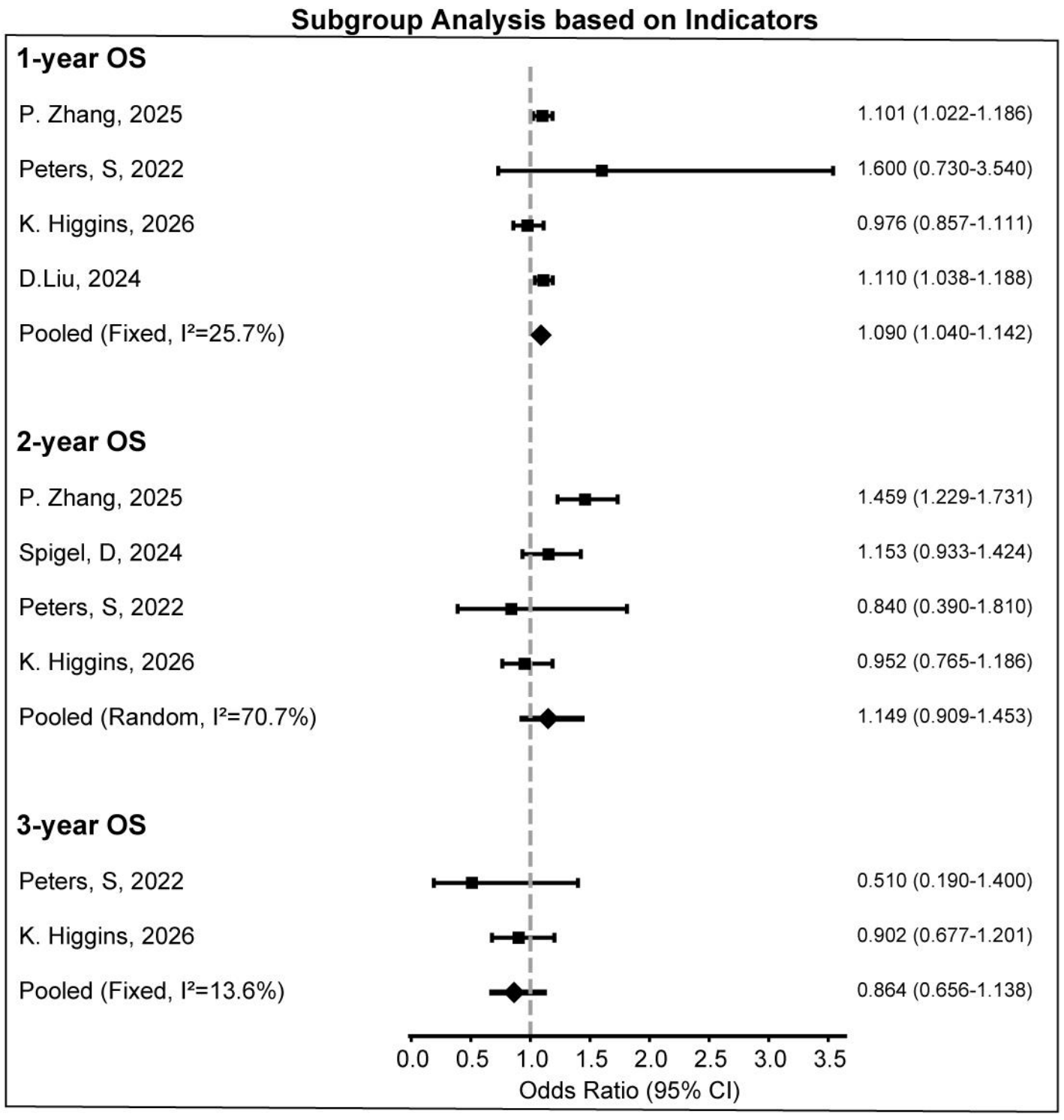

Fig. S2. 1-year, 2-year and 3-year OS rate.

Fig. S3.

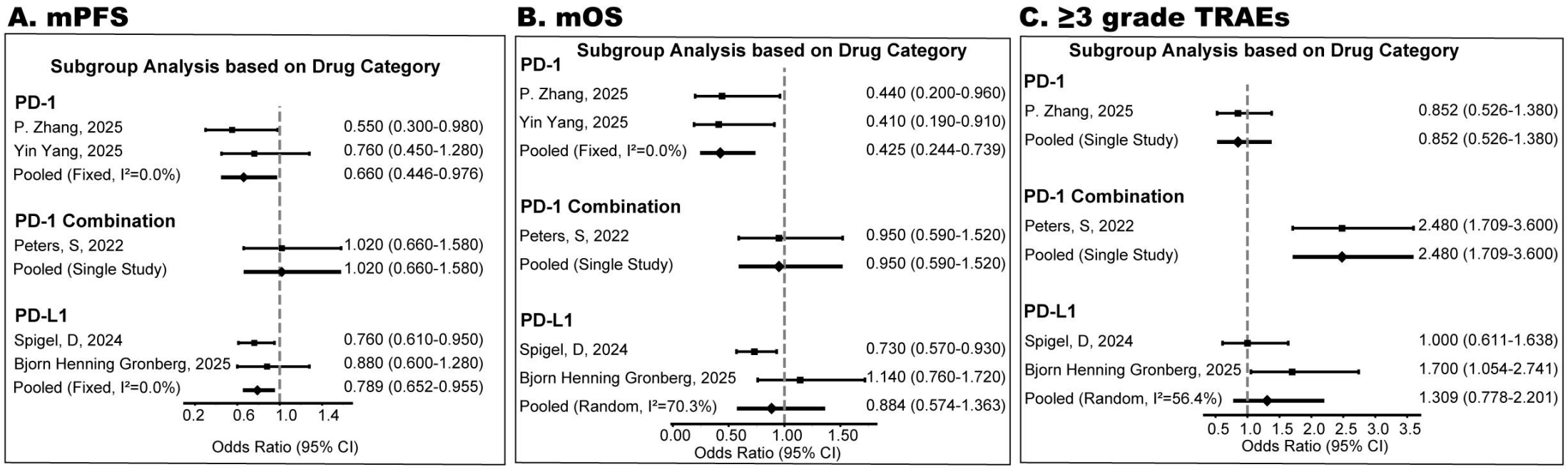

Fig. S3. Evaluation on mPFS, mOS and ≥3 grade TRAEs in the consolidation group.

Fig. S4.

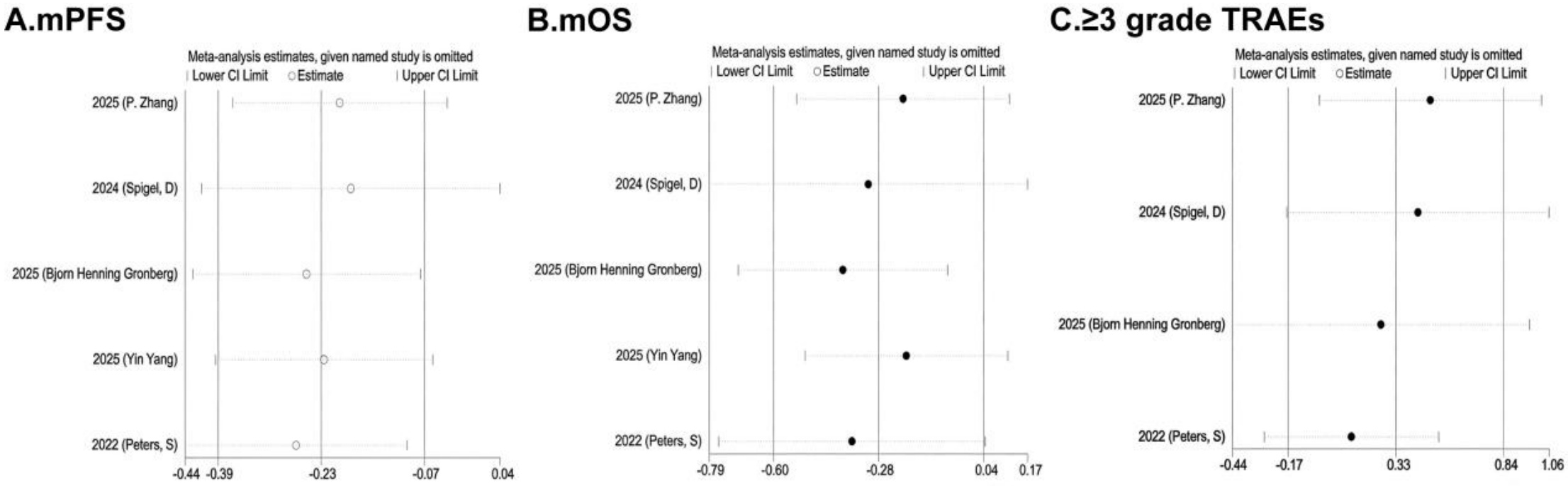

Fig. S4. Sensitivity analysis of mPFS, ORR and ≥3 grade TRAEs in the consolidation group.

Fig. S5.

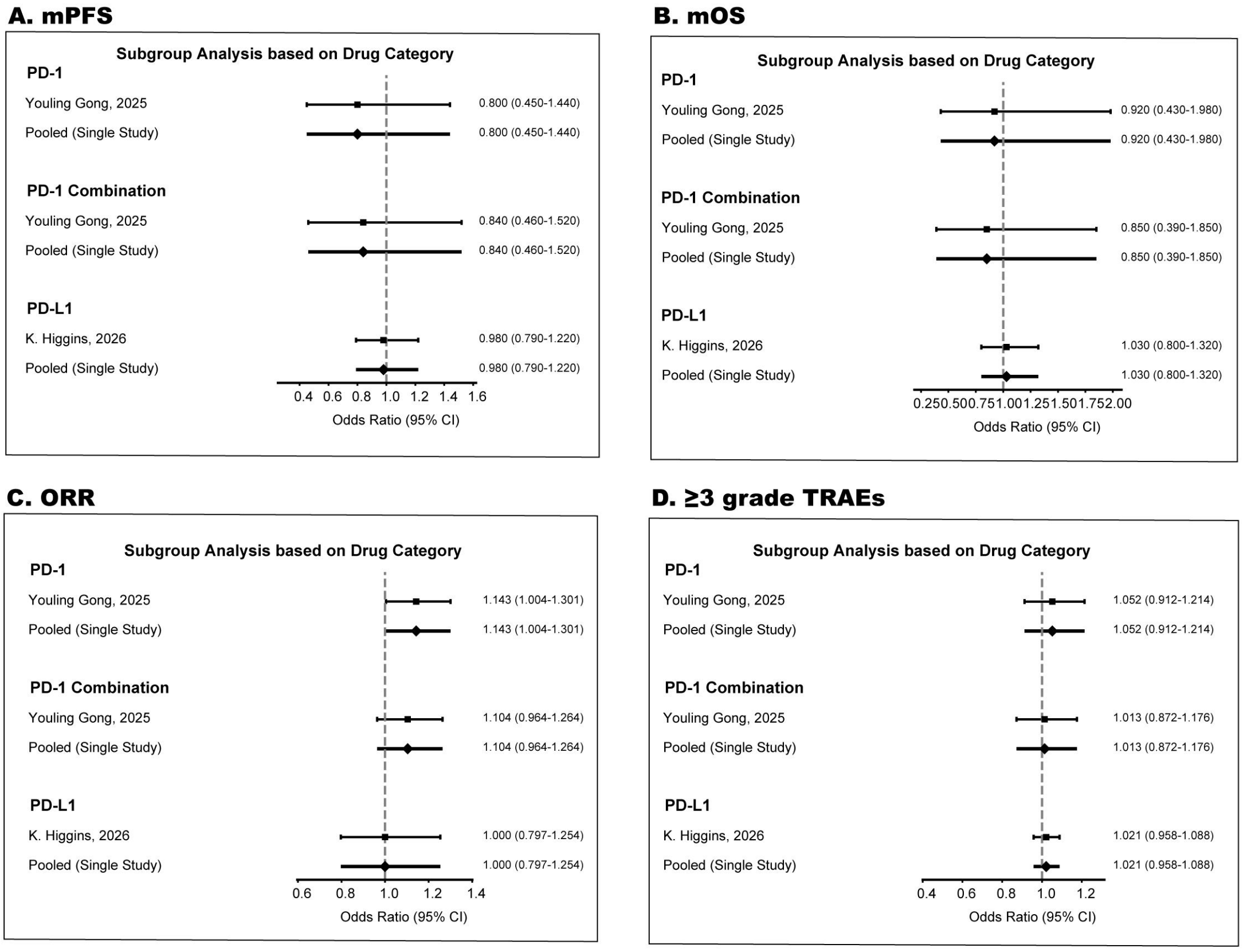

Fig. S5. Evaluation on mPFS, mOS, ORR and ≥3 grade TRAEs in the concurrent+consolidation group.

Fig. S6.

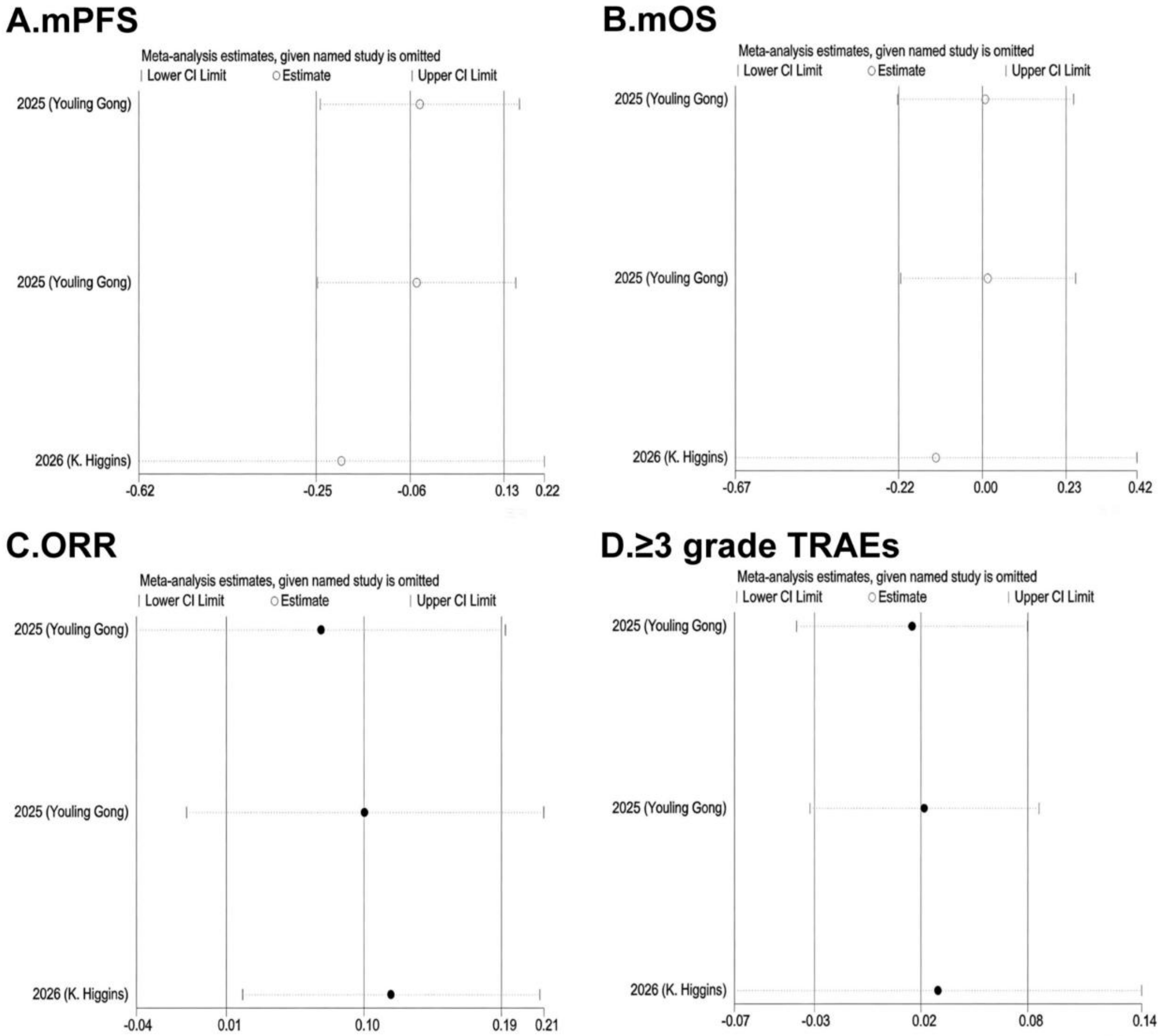

Fig. S6. Sensitivity analysis of mPFS, mOS, ORR and ≥3 grade TRAEs in the concurrent+consolidation group.

Fig. S7.

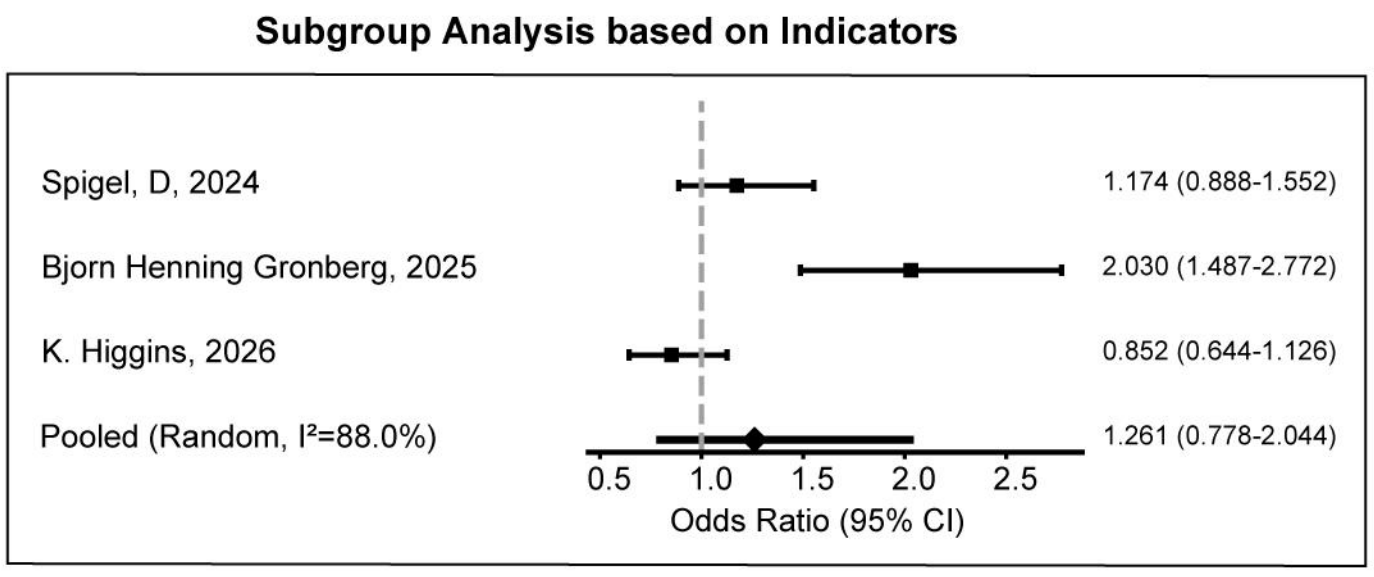

Fig. S7. Impact of PCI in immunotherapy.
